# Temperature, ozone, and mortality in urban and non-urban counties in the northeastern United States

**DOI:** 10.1186/1476-069X-14-3

**Published:** 2015-01-07

**Authors:** Jaime Madrigano, Darby Jack, G Brooke Anderson, Michelle L Bell, Patrick L Kinney

**Affiliations:** Department of Environmental and Occupational Health, School of Public Health, Rutgers, The State University of New Jersey, Piscataway, NJ USA; Department of Environmental Health Sciences, Mailman School of Public Health, Columbia University, New York, NY USA; Department of Environmental and Radiological Health Sciences, Colorado State University, Fort Collins, CO USA; School of Forestry and Environmental Studies, Yale University, New Haven, CT USA

**Keywords:** Climate change, Epidemiology, Mortality, Ozone, Time-series models

## Abstract

**Background:**

Most health effects studies of ozone and temperature have been performed in urban areas, due to the available monitoring data. We used observed and interpolated data to examine temperature, ozone, and mortality in 91 urban and non-urban counties.

**Methods:**

Ozone measurements were extracted from the Environmental Protection Agency’s Air Quality System. Meteorological data were supplied by the National Center for Atmospheric Research. Observed data were spatially interpolated to county centroids. Daily internal-cause mortality counts were obtained from the National Center for Health Statistics (1988–1999). A two-stage Bayesian hierarchical model was used to estimate each county’s increase in mortality risk from temperature and ozone. We examined county-level associations according to population density and compared urban (≥1,000 persons/mile^2^) to non-urban (<1,000 persons/mile^2^) counties. Finally, we examined county-level characteristics that could explain variation in associations by county.

**Results:**

A 10 ppb increase in ozone was associated with a 0.45% increase in mortality (95% PI: 0.08, 0.83) in urban counties, while this same increase in ozone was associated with a 0.73% increase (95% PI: 0.19, 1.26) in non-urban counties. An increase in temperature from 70°F to 90°F (21.2°C 32.2°C) was associated with a 8.88% increase in mortality (95% PI: 7.38, 10.41) in urban counties and a 8.08% increase (95% PI: 6.16, 10.05) in non-urban counties. County characteristics, such as population density, percentage of families living in poverty, and percentage of elderly residents, partially explained the variation in county-level associations.

**Conclusions:**

While most prior studies of ozone and temperature have been performed in urban areas, the impacts in non-urban areas are significant, and, for ozone, potentially greater. The health risks of increasing temperature and air pollution brought on by climate change are not limited to urban areas.

**Electronic supplementary material:**

The online version of this article (doi:10.1186/1476-069X-14-3) contains supplementary material, which is available to authorized users.

## Introduction

Ground level ozone and temperature are current environmental health stressors that are expected to worsen with climate change. Daily mortality is associated with short-term peaks in both ozone and temperature [1–6], which often co-occur during warm months. However, one recent study found that the association between ozone and mortality depends on the specification of temperature in the model [7]. Since the formation of ozone is temperature dependent, ozone may or may not be included in models that assess temperature-related mortality, depending on the research question [8].

Additionally, environmental data are often rich in time but sparse in space hampering health effects analysis and leading to generalizations from studies where data are available. Most health effects studies of ozone and temperature have been performed in urban areas [1, 9–11] and very limited work has been done in suburban and rural areas [12–14] with conflicting results as to whether the magnitude of risk is the same in urban and non-urban areas. The disparity in number of research studies for urban and non-urban areas relates to the nature of large-scale monitoring networks for ozone monitors, which are more likely to be in urban areas, and concerns of population size, which are less problematic for studies focusing on urban populations. Similarly, many public health preparedness efforts for climate change adaptation (e.g. heat-health warning systems) have been concentrated in major metropolitan areas [15] and there is some evidence that rural communities are not well represented in climate and health research [16]. Although, several recent studies have suggested that heat-health risks are also of concern in rural areas [17–19]. If non-urban areas are also at risk, local public health departments may be ill equipped to deal with the health impacts of a changing climate, or even to develop efficient policies to mitigate the current risks. Research results from one area may not be applicable to another due to differences in population demographics, health care systems, baseline health care status, and other factors [20–22].

In this study, we used a combination of observed and interpolated data to examine the relationship between temperature, ozone, and mortality in 91 counties in the Northeastern United States, comprising urban, suburban, and rural areas. We wanted to determine how the use of interpolated data influenced our results, compared to those using observed data alone. We also wanted to evaluate whether the risk was the same in urban versus non-urban counties in our study area, given the paucity of information on health risks of air pollution in non-urban regions and since assessing these differences within a given geographical region remains an understudied area of research. We hypothesized that the risks in non-urban areas would be at least as great as those in urban areas. Finally, we assessed whether county characteristics could explain variation in the county-level associations.

## Methods

We obtained daily mortality records from the National Center for Health Statistics for every county in the states of New York, New Jersey, and Connecticut. There are 62 counties in New York, 21 counties in New Jersey, and 8 counties in Connecticut, for a total of 91 counties. Data were obtained for the years 1988 through 1999. Daily mortality counts were computed by adding all deaths from internal causes (i.e., non-accidental deaths) that occurred on each day in each county. We performed this analysis in two phases; first, using only observed (i.e., measured) ozone and temperature data, and second, incorporating interpolated data.

### Exposure

Ozone measurements were extracted from EPA’s AQS (Air Quality System) for all stations in the eastern U.S. from 1988 – 1999. For ozone, the daily maximum 8-hour average was derived from hourly ozone data obtained from AQS and matched to a master station file obtained from EPA. A daily mean 8-hour ozone value from all ozone-monitoring stations in each county was calculated. We restricted attention to 12 counties (2 in Connecticut, 2 in New Jersey, and 8 in New York) for which both ozone and temperature data were available for at least 75% of possible days. Ten counties met these criteria for the entire study period (1988 – 1999). For two counties (Chautauqua, NY and New Haven, CT), the study period was restricted to years that met the criteria, 1992 – 1999 and 1993 – 1999, respectively. We obtained meteorological data (daily maximum temperature) from the CISL Research Data Archive (http://rda.ucar.edu/datasets/ds472.0/).

In the second phase of this analysis, observed ozone and temperature data from all sites within a rectangular domain that encompassed the tri-state area were spatially interpolated (kriging) to the 91 population-weighted county centroids (this is illustrated by Additional file 1: Figures S1 and S2). We first estimated variograms for each day for each variable. Maximum likelihood fitting was then used to fit five alternative theoretical variogram models (circular, spherical, cubic, Gaussian, and exponential) to the empirical variogram. The best fit was determined for each day and variable by cross-validation and maximum likelihood goodness of fit metrics. Using this approach, data were interpolated to population-weighted county centroids. Population-weighted centroids were computed as the point in each county that minimized the sum of squared distances to the people living there. This method generates a centroid that represents the average center of persons’ residences in the county, as opposed to area-weighting, which notes the center of the area. Thus, this method accounts for differences in population density throughout the county.

### Statistical analysis

We performed this analysis in two phases. In the first phase, we used data only from counties (n = 12) with observed environmental data for at least 75% of available days in a given year for at least 5 years. In the second phase, we incorporated interpolated exposure data for all 91 counties for all possible days. During each phase, we applied a two-stage Bayesian hierarchical model. In the first stage, we estimated increase in mortality risk using generalized linear models with an over-dispersed Poisson distribution for each county. We ran separate models to estimate the effects of daily 8-hour maximum ozone and daily maximum temperature. When assessing the effect of temperature, we ran models without control for ozone and included ozone as a covariate as a sensitivity analysis. When assessing the effect of ozone, our primary analysis included temperature as a covariate. In all models, we controlled for day of the week and time trends, to account for seasonal and long-term time trends. The general structure of each county-specific model was:
1

where


We estimated a separate effect for each county using equation 1 and then generated an overall effect estimate across all counties by combining the county-specific effects, accounting for the estimate’s statistical uncertainty, using a Bayesian hierarchical model through the Two-Level Normal independent sampling estimation (TLNise) package in R [23]. Similar community-specific models and Bayesian hierarchical modeling strategies were used in previous work to analyze how ozone and temperature are associated with mortality risk [20, 24]. We calculated the relative increase in mortality for a 10 ppb increase in daily 8-hour maximum ozone. To quantify results from the non-linear function relating temperature to risk of mortality, we estimated a heat effect by comparing the increase in mortality at 90°F, corresponding to approximately the 99^th^ percentile of the temperature distribution of all counties, versus 70°F (32.2°C versus 21.1°C).

Due to data availability during the study period at the county level, we were unable to account for other environmental pollutants (e.g., particulate matter) that may be correlated with ozone and associated with daily mortality. However, in one county (New Haven, CT) where consistent PM_10_ data were available throughout the study period, we performed a sensitivity analysis to determine if adjustment for PM_10_ affected the association between ozone and mortality.

We next compared the estimates between urban and non-urban counties. We stratified our data by population density, defining urban counties as those having a population density greater than or equal to 1,000 persons per square mile and non-urban counties as those having a population density of less than 1,000 persons per square mile (one dimension used by the U.S. Bureau of the Census to define urban). Population density estimates were obtained from the year 2000 US Census, and a sensitivity analysis used data from the 1990 Census. We then computed two summary estimates; one for the urban counties (n = 23) and one for the non-urban counties (n = 68).

Finally, to determine if heterogeneity between counties could be explained by population characteristics, we fit the following Bayesian hierarchical regression model:


where


The following county characteristics were obtained from the United States Census summary files: proportion of county families living in poverty, population density, and proportion of residents over the age of 65. We also investigated whether mean county ozone concentration or temperature modified the county-specific results. All of our analyses were restricted to warm months (April – October) as this time of the year has the highest levels of our exposures of interest (ozone and temperature). Analyses were conducted using R 2.8.1 (R Foundation for Statistical Computing, Vienna, Austria).

## Results

Summary statistics for mortality, ozone, and temperature for each of the 12 counties used in the analysis with observed data, as well as summaries for these 12 counties, all 91-study counties, 23 urban counties, and 68 non-urban counties, are shown in Table 1. Across all 91 counties, the mean observed daily 8-hour maximum ozone concentration was 46 ppb and the mean warm-month daily maximum temperature was 74°F. On average, there were 6 deaths per day (range 0 to 99) across the 91 counties.

**Table 1:** **Environmental and mortality data for study area, April – October, 1988 - 1999**

	Ozone (ppb)			Maximum Temperature (°F)			Mortality (n/day)		
	Minimum	Maximum	Mean	Minimum	Maximum	Mean	Minimum	Maximum	Mean
Hartford, CT	0	146	44.3	33.5	100.5	73.4	5	34	17.4
New Haven, CT	4	127	44.6	32	97	71.1	7	36	17.2
Atlantic, NJ	3	136	53.8	41	100	74.4	0	15	5.2
Essex, NJ	1	145	37.1	40	105	75.8	4	40	17.6
Albany, NY	0	116	42.8	30	98	71.2	0	18	6.1
Chautauqua, NY	10	107	49.9	24	93	66.3	0	11	3.2
Chemung, NY	4	118	45.3	34	102	71.8	0	9	2.2
Erie, NY	1	134	44.8	31	96	60.1	10	45	24.4
New York, NY	3.5	149	45.4	39	102	74.8	11	67	34.3
Niagara, NY	6	134	46.5	30	99	70.1	0	15	4.8
Suffolk, NY	8	138	47.7	37	101	71.8	11	47	23.9
Westchester, NY	3	150	45.2	34	100	72.5	3	33	17.0
12 Counties (using observed data)	1	149	45.6	29	104	71.8	0	67	14.4
91 Counties (using kriging data)	4.7	136.6	45.7	35.2	102.4	73.8	0	99	6.2
23 Urban Counties	4.7	133.5	45.6	40.7	102.4	75.8	0	99	17.2
68 Non-Urban Counties	5.3	136.6	45.7	35.2	100.0	73.1	0	45	2.5

### Observed data analysis

**Figure 1:**
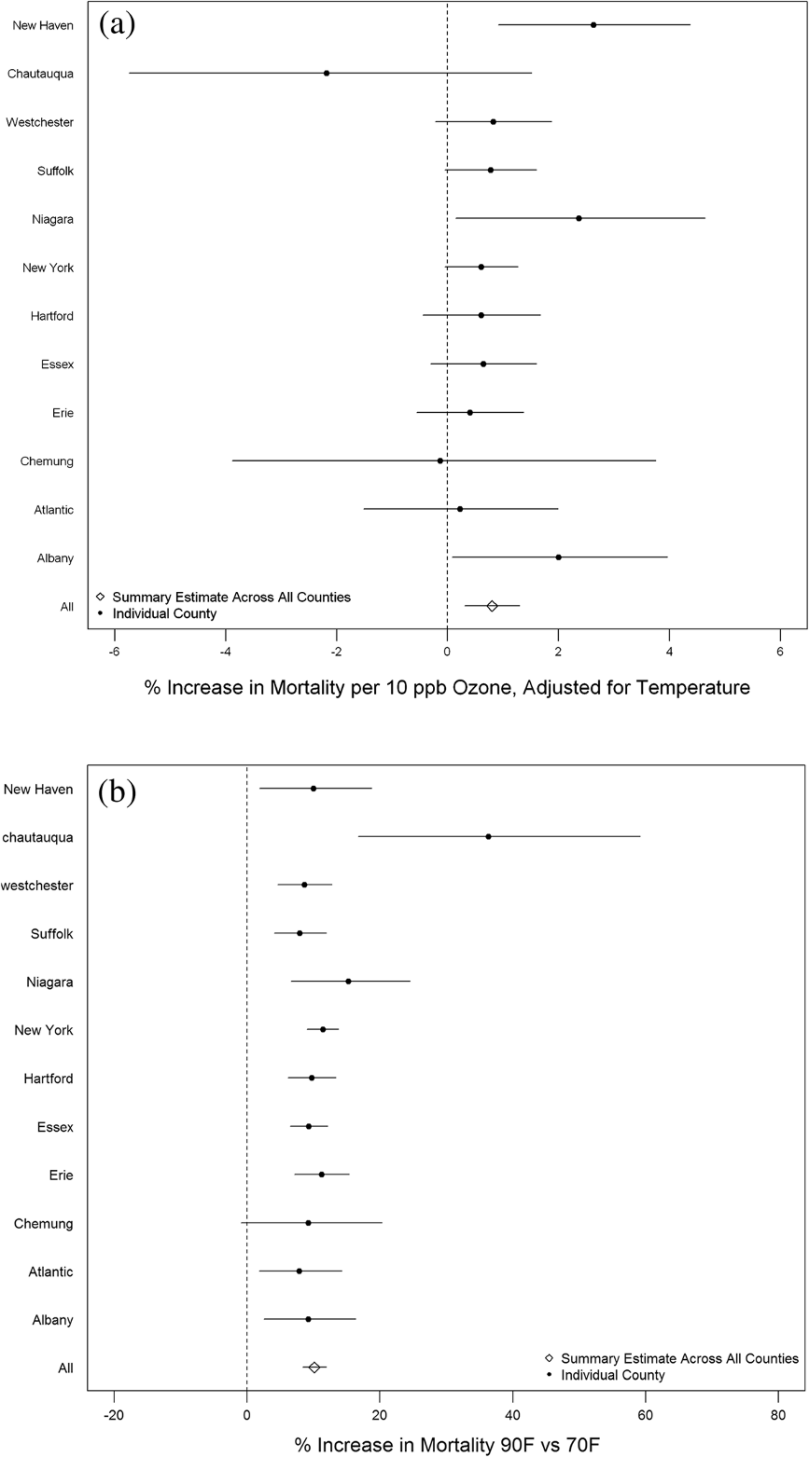
**Point estimates and 95% posterior intervals showing association between (a) ozone and mortality in twelve counties and (b) temperature and mortality in twelve counties.**

In the analysis of 12 counties with observed data, we found that an increase in exposure to ozone was associated with an increase in risk of mortality in the majority of counties (Figure 1a). When we generated an overall effect across counties by combining county-specific effect estimates, we found that a 10 ppb increase in daily 8-hour maximum ozone was associated with a 0.80% increase in mortality [95% Posterior Interval (PI): 0.31, 1.30], after adjusting for temperature. Similarly, we found that mortality was statistically significantly increased in all but one county when comparing days of 90°F versus 70°F (Figure 1b). Across counties, we found that there was an increase in mortality of 10.11% (95% PI: 8.34, 11.91) when comparing expected mortality at 90°F versus 70°F.

### Kriging data analysis

**Figure 2:**
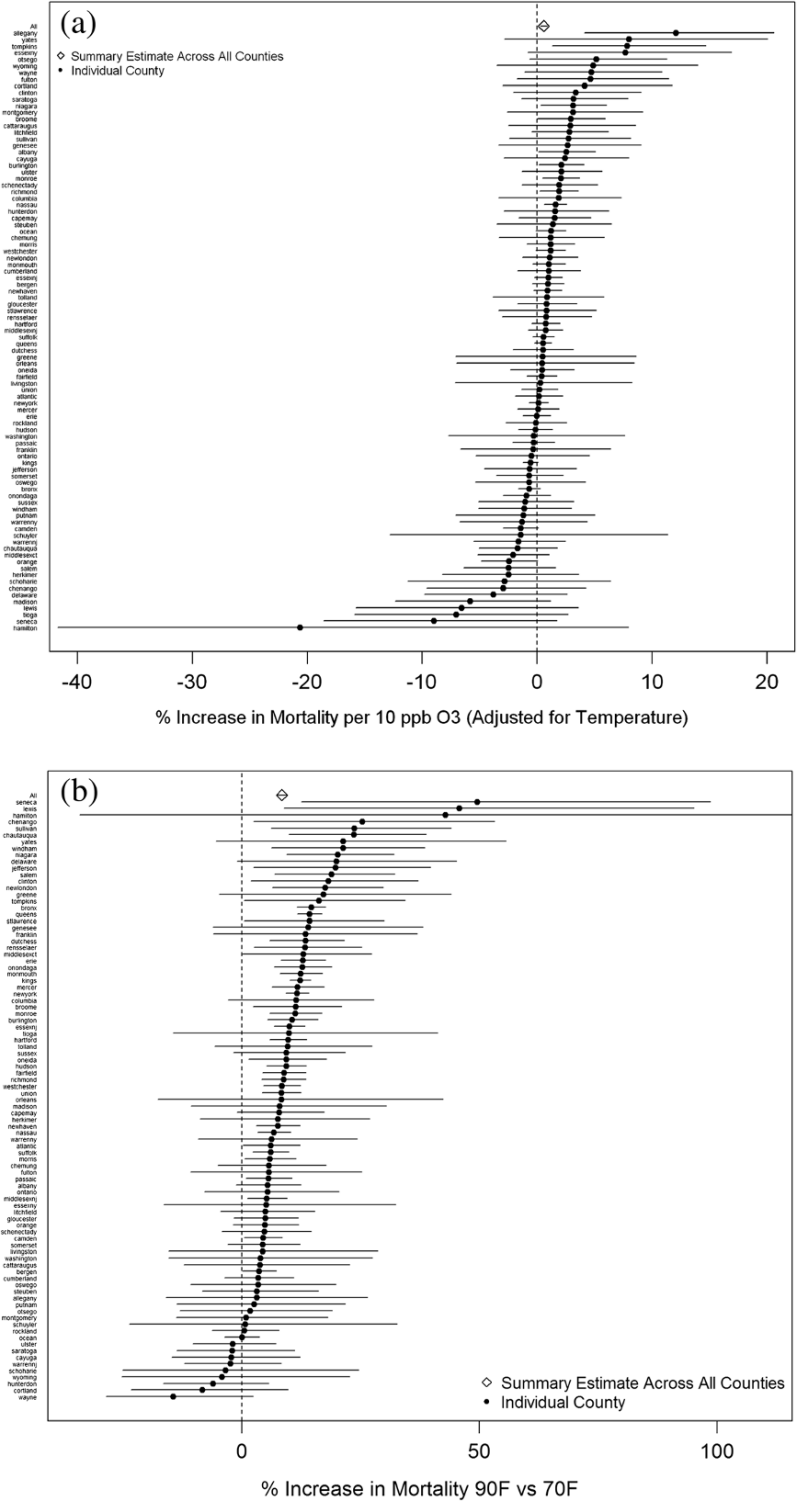
**Point estimates and 95% posterior intervals showing association between (a) ozone and mortality in 91 counties and (b) temperature and mortality in 91 counties.**

In our analysis of 91 counties using data from kriging methods, we found some variability in the effect estimates for ozone and temperature (Figure 2a and b), but overall, across counties, we found a 10 ppb increase in daily 8-hour maximum ozone was associated with a 0.55% increase in mortality (95% PI: 0.25, 0.86), after adjustment for temperature. There was an increase in mortality of 8.44% (95% PI: 7.24, 9.65) when comparing expected mortality at 90°F versus 70°F.

### Sensitivity analysis

In our models for the observed data we used a spline with 7 degrees of freedom per year to control for time trends, however, due to model convergence problems when using data for all 91 counties, we reduced the degrees of freedom to 4 per year for the 91-county analysis. To assess how this would impact our results, we re-ran the models for only the 12 counties with observed data, but using 4 degrees of freedom per year. A comparison of the results across all counties from these three methods is shown in Table 2. We found that using 4 degrees of freedom instead of 7 degrees of freedom did not substantially impact the results.

**Table 2:** **Summary estimates across counties**

	Increase in Mortality for a 10 ppb Increase in Ozone				Increase in Mortality Comparing 90°F to 70°F			
	Unadjusted		Adjusted for temperature		Unadjusted		Adjusted for ozone	
Data	% Increase	PI	% Increase	PI	% Increase	PI	% Increase	PI
12 counties, 7 df	1.54	(1.19,1.90)	0.80	(0.31,1.30)	10.11	(8.34,11.91)	6.67	(4.13,9.27)
12 counties, 4 df	1.50	(1.14,1.86)	0.70	(0.10,1.22)	9.63	(7.42,11.89)	6.39	(2.93,9.97)
91 counties, 4 df	1.39	(1.22,1.57)	0.55	(0.25,0.86)	8.44	(7.24,9.65)	5.87	(3.95,7.82)

ppb parts per billion.

PI posterior interval.

df degrees of freedom per year for spline of time in model.

Although we did not have complete data for particulate matter for all counties during our study period, we ran a sensitivity analysis for New Haven county (CT), where sufficient PM_10_ data were available. Without adjustment for PM_10_, there was a 2.54% (95% PI: 0.78, 4.33) increase in mortality for a 10 ppb increase in daily 8-hour maximum ozone in New Haven. After adjustment for PM_10_, there was a 2.49% (95% PI: 0.69, 4.31) increase in mortality for a 10 ppb increase in daily 8-hour maximum ozone in New Haven.

### Urban and non-urban analysis

**Figure 3:**
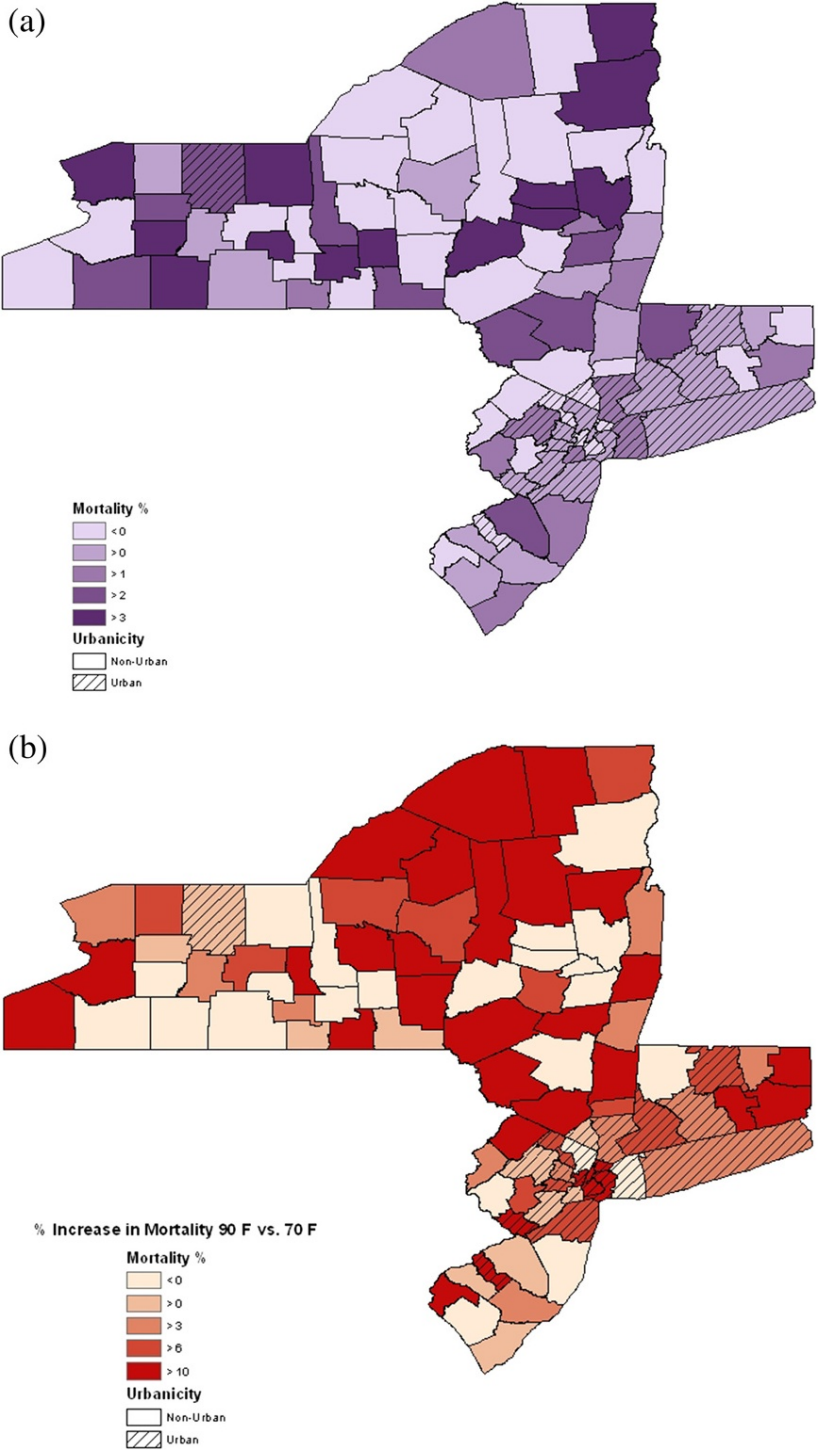
**Percent increase in mortality by county for (a) a 10 ppb increase in ozone and (b) comparing 90°F to 70°F.**

When we stratified our data into urban and non-urban counties, the effect of ozone, after adjusting for temperature, in urban counties was a 0.45% (95% PI: 0.08, 0.83) increase in mortality for a 10 ppb increase in daily 8-hour maximum ozone. Across non-urban counties, there was a 0.73% (95% PI: 0.19, 1.26) increase in mortality for a 10 ppb increase in daily 8-hour maximum ozone. When assessing the impact of temperature across urban counties, there was an 8.88% (95% PI: 7.38, 10.41) increase in mortality comparing expected mortality at 90°F to 70°F. Across non-urban counties, this estimate was 8.08% (95% PI: 6.16, 10.05). Figure 3a and b show the variation in these estimates by county and according to urban or non-urban classification. A sensitivity analysis using population density estimates from the 1990 US Census did not impact these results.

### County characteristic effect modification

Urban counties had a higher mean population density, proportion of families living in poverty, mean temperature, and lower proportion of residents over the age of 65 (Table 3). Table 4 shows the results of the effect modification analysis, including the overall relative increase in mortality after adjusting for the county characteristic and the percentage change in the mortality effect estimate due to each county characteristic. The percent of families living in poverty and population density both modified the association of temperature and ozone with mortality. With increasing levels of these characteristics, there was a greater association between temperature and mortality and a lower association between ozone and mortality. The percentage of residents over 65 in a county only explained heterogeneity of the ozone estimate, which increased with a greater number of residents over 65. Variation in mean temperature and ozone in a county did not explain any of the heterogeneity observed in our results.

**Table 3:** **County characteristics by urban and non-urban classification**

	Urban counties (n = 23)	Non-Urban counties (n = 68)
	Mean ± SD	Mean ± SD
% Families in poverty	8.9 ± 6.3	7.2 ± 2.6
Population density, person/mile^2^	9,390.4 ± 15,756.8	231.3 ± 224.8
% Over 65	12.7 ± 1.3	14.0 ± 2.6
Ozone, ppb	45.6 ± 2.5	45.7 ± 2.8
Temperature, °F	75.8 ± 1.4	73.1 ± 2.0

**Table 4:** **Modification by county characteristics**

	% Increase in mortality per 10 ppb ozone, adjusted for community characteristic		% Change in ozone effect estimate per IQR ^1^ increase in community level variable		% Increase in mortality 90 F vs 70 F, adjusted for community characteristic		% Change in temperature effect estimate per IQR ^1^ increase in community level variable	
	Central estimate	95% PI	Central estimate	95% PI	Central estimate	95% PI	Central estimate	95% PI
No modification by county characteristic	0.55	(0.25, 0.86)	-	-	8.44	(7.24, 9.65)	-	-
% Families Poverty	0.64	(0.37, 0.90)	−0.02	(−0.04, −0.01)	8.21	(7.17, 9.26)	1.22	(0.59, 1.85)
Population Density	0.66	(0.36, 0.96)	−0.002	(−0.003,−0.0001)	8.09	(6.94, 9.26)	0.08	(0.01, 0.15)
% Over 65	0.59	(0.31, 0.88)	4.41	(0.75, 8.21)	8.37	(7.20, 9.55)	−51.42	(−88.29, 101.45)
Mean ozone	0.56	(0.25, 0.86)	0.001	(−0.02, 0.02)	8.60	(7.45, 9.76)	−0.71	(−1.51, 0.09)
Mean temperature	0.70	(0.35, 1.05)	−0.04	(−0.10, 0.01)	9.02	(7.66, 10.40)	−1.81	(−3.78, 0.21)

^1^IQR = Interquartile Range; IQR for families in poverty = 3.6%, IQR for population density = 891 persons/mile^2^, IQR for residents over age 65 = 2.9%, IQR for ozone concentration = 2.3 ppb, IQR for temperature = 3.6°F.

All ozone results include adjustment for temperature; temperature results do not include adjustment for ozone. Estimates are based on 4 degrees of freedom/year for temporal trend.

## Discussion

In this analysis of 91 counties in the Northeastern United States, we found that ozone and temperature were independently associated with daily mortality. Several studies have shown an association between ozone and all-cause mortality [1, 2, 25] and our estimate of the association between ozone on mortality is consistent with that seen in a study of 95 urban communities in the US [1]. Similarly, our finding of an association between heat and all-cause mortality is consistent with the literature [26].

Recently, there has been a discussion in the literature of the most appropriate way to adjust for temperature when examining ozone-related mortality, as well as the appropriateness of controlling for ozone in studies of temperature-related mortality. Pattenden and colleagues found that their estimate of ozone-related mortality was most sensitive to adjustment by temperature when maximum daily temperature was used [7], and therefore, we present results for ozone adjusted for maximum daily temperature. In assessing temperature-related mortality, some studies adjust for ozone and others do not. As Reid and colleagues outline, ozone can cause mortality, but it is unlikely to have a sufficient impact on local temperature to cause a health response [8]. Therefore, ozone can be thought of as a causal intermediate in the relationship between temperature and mortality, but is unlikely to confound the association between temperature and mortality. We primarily present the results of models examining temperature-related mortality, unadjusted for ozone, but also show these results adjusted for ozone. The former can be thought of as the total effect of temperature on mortality, including through any increases in ozone concentration it causes, while the latter can be thought of as the controlled direct effect of temperature on mortality.

In this study we used a combination of measured and interpolated data to assess exposure across three states in the Northeast. Although more extensive methods of estimating air pollution levels are available, we found that a method of interpolation provided useful information, and future studies may investigate other approaches to estimate ozone levels. By incorporating additional estimates of ozone into our analysis, we were able to evaluate the health impacts of ozone and temperature in every county across the tri-state region, including those without monitors. Additionally, we were able to generate an overall effect estimate using more information throughout the region (91 as opposed to 12 counties), as well as examine differences in urban and non-urban counties. Finally, by using this expanded set of counties, and matching it to publicly available data, we were able to examine a set of population characteristics as explanatory variables for the observed variation in results.

Previous large studies in the US, have estimated that a 10 ppb increase in ozone contributes to an increased risk of mortality of between 0.39% (95% CI: 0.26, 0.51) [2] and 0.52% (95% PI: 0.27, 0.77) [1], which is consistent with our overall result of a 0.55% (95% PI: 0.25, 0.86) increase. However, when we stratified counties by a measure of urbanicity (above or below 1,000 persons/mile^2^), we found that the point estimate for the association between ozone and mortality was higher for non-urban counties. Although the confidence intervals between the two estimates overlapped, the central estimate for non-urban counties was 1.6 times greater than the estimate for urban counties. Since most prior investigations of ozone and mortality have been conducted in urban areas, this may indicate that the public health burden of ozone is even greater than previously estimated. Our assessment of the impact of temperature found that significant associations between temperature and mortality persisted in both urban and non-urban counties. This indicates that the health impacts of temperature are not limited to the “urban heat island”. Few other studies in the U.S. have examined temperature-related mortality and morbidity across urban, suburban, and rural areas, but there are some indications that being an urban resident does not necessarily make one more vulnerable to heat [14, 19].

Studies indicate that many local governments do not feel adequately prepared to address the health impacts of heat [27] and, specifically, in a survey of local health departments in New York state, the majority of respondents did not feel that their department had the necessary expertise to assess potential public health risks of climate change in their jurisdiction [28]. New York, New Jersey, and Connecticut include both urban and non-urban areas and the decentralized public health structure, along with limited budgets, can mean that public health preparedness for climate change can be a challenge. Our findings indicate that both urban and non-urban areas should be prepared to address this challenge.

When we examined county characteristics that might explain the observed variation in our effect estimates, we found that greater poverty in a county was associated with larger effect estimates for heat and mortality. This is consistent with other studies that have examined modification in the temperature-mortality association. Both individual and neighborhood characteristics related to lower socioeconomic position have been found to enhance the relationship between temperature and mortality [10, 29, 30], and this may be partially explained by lack of financial resources to keep indoor environments cool or poorer baseline health. On the contrary, the inverse association of poverty observed for the ozone and mortality association is not consistent with prior investigations that provide evidence for an enhanced impact of ozone among populations with higher unemployment [20, 31]. Whether our findings are indicative of greater/lesser susceptibility among impoverished populations or they serve as a marker for other unobserved characteristics associated with urban populations, similar to population density, cannot be distinguished from this data. In our data, a greater proportion of residents over age 65 in a county was associated with increased risk of mortality from ozone, and this is consistent with several studies that have showed that the elderly are more susceptible to the health impacts of ozone and that age is one of the strongest risk factors for ozone sensitivity [31, 32]. Neither mean concentration of ozone nor mean temperature served to explain variation in the observed effect estimates.

Our analysis did have a number of limitations. We used one estimate of ozone and temperature for each county, which could have contributed to error in our exposure estimate. Our analysis was also restricted to natural causes of death, potentially underestimating the effect of temperature, as heat stroke is classified as an external cause of death. Due to data availability during the study period at the county level, we were unable to account for other environmental pollutants (e.g., particulate matter) that may also be correlated with ozone and associated with daily mortality. While some studies in the region have shown confounding of the ozone mortality association by other pollutants [33, 34], other national studies have shown that the ozone mortality association is robust [1, 2]. In our data for one county (New Haven, CT), we found no significant difference in our model results for the effect of ozone after adjusting for PM_10_. Our investigation of effect modification was based on county-level data and not individual-level data. Additionally, our examination of county characteristics was based on publicly available data and, therefore, not exhaustive. Other factors, including baseline health, activity patterns, and other population characteristics, may also account for the variation between county effect estimates that was observed. Our novel finding of a greater magnitude of association between ozone and mortality in non-urban counties than in urban counties requires replication. However, if further work can substantiate these findings it may indicate that the public health burden of ozone has been underestimated and current regulatory standards are not fully protective.

## Conclusions

We found effects of both ozone and temperature on mortality in this county-level analysis of three states in the Northeastern United States. By making use of a simple method of interpolation, we were able to estimate these associations over a wider geographic area and population than previous investigated. We also found that these associations existed in both urban and non-urban counties, with a potentially greater association between ozone and mortality in non-urban areas than in urban areas. These results indicate that the health risks brought on by climate change are not limited to urban areas and warrant further investigation.

## Electronic supplementary material

Additional file 1: Figure S1: Example of kriging of 8-hour maximum ozone concentrations for July 18, 1999 within the northeastern US domain. **Figure S2.** Example of kriging of daily maximum temperature for July 18, 1999 within the northeastern US domain. (PDF 115 KB)

## Abbreviations

AQS: Air quality system
EPA: Environmental protection agency
ppb: Parts per billion
PI: Posterior interval.
