# Supplementary material for: Temperature, ozone, and mortality in urban and non-urban counties in the northeastern United States
Source: Environ Health. 2015 Jan 7;14:3. doi: 10.1186/1476-069X-14-3 (PMC4417233; doi:10.1186/1476-069X-14-3)

Figure S.1. Example of kriging of 8-hour maximum ozone concentrations for July 18, 1999 within the northeastern US domain. For the health analysis, we used data kriged to the population-weighted county centroids in NY, NJ, and CT, as shown in the lower left hand panel.

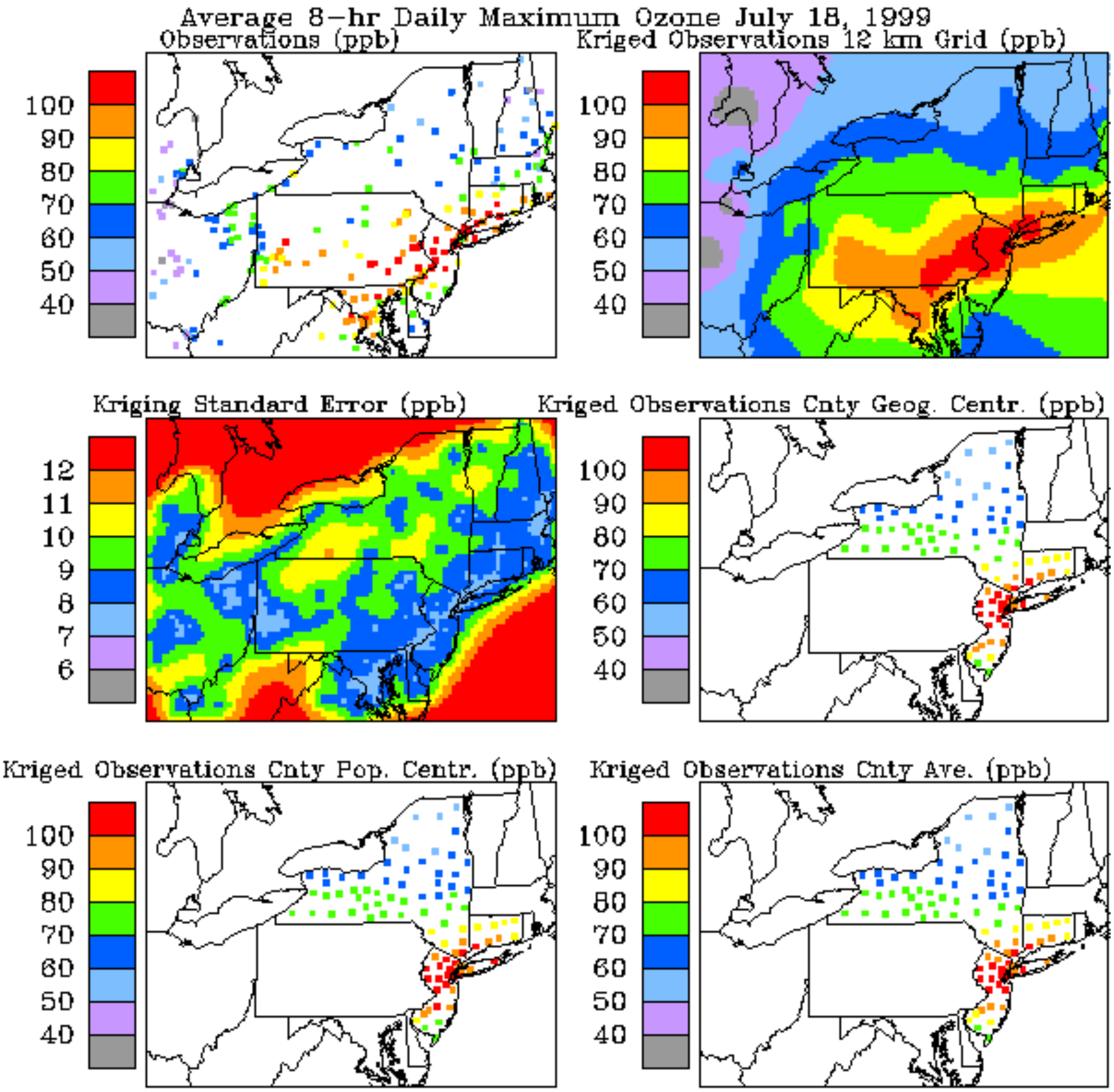

Figure S.2. Example of kriging of daily maximum temperature for July 18, 1999 within the northeastern US domain. For the health analysis, we used data kriged to the population-weighted county centroids in NY, NJ, and CT, as shown in the lower left hand panel.

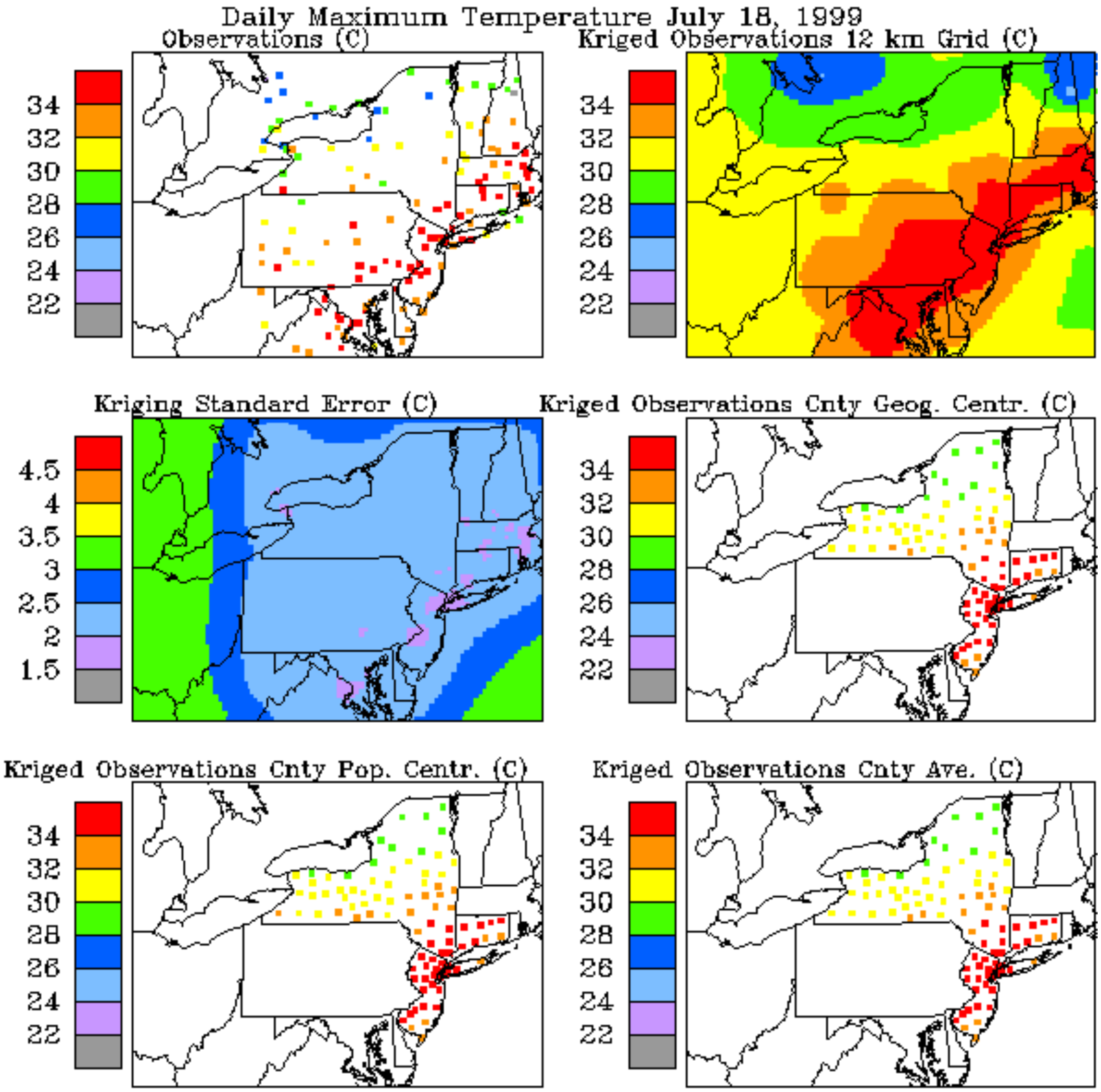

Supplement: Supplementary file 1 — Additional file 1: Figure S1: Example of kriging of 8-hour maximum ozone concentrations for July 18, 1999 within the northeastern US domain. Figure S2. Example of kriging of daily maximum temperature for July 18, 1999 within the northeastern US domain. (PDF 115 KB) [file 12940_2014_840_MOESM1_ESM.pdf]
